# Novel image features of optical coherence tomography for pathological classification of lung cancer: Results from a prospective clinical trial

**DOI:** 10.3389/fonc.2022.870556

**Published:** 2022-10-21

**Authors:** Qiang Zhu, Hang Yu, Zhixin Liang, Wei Zhao, Minghui Zhu, Yi Xu, Mingxue Guo, Yanhong Jia, Chenxi Zou, Zhen Yang, Liangan Chen

**Affiliations:** ^1^ Department of Respiratory Medicine, The First Medical Center of Chinese People Liberation Army (PLA) General Hospital, Beijing, China; ^2^ Department of Pulmonary and Critical Care Medicine, Zhongnan Hospital of Wuhan University, Wuhan, China

**Keywords:** bronchoscopy, lung cancer, OCT, AFB, WLB

## Abstract

**Background:**

This study aimed to explore the characteristics of optical coherence tomography (OCT) imaging for differentiating between benign and malignant lesions and different pathological types of lung cancer in bronchial lesions and to preliminarily evaluate the clinical value of OCT.

**Methods:**

Patients who underwent bronchoscopy biopsy and OCT between February 2019 and December 2019 at the Chinese PLA General Hospital were enrolled in this study. White-light bronchoscopy (WLB), auto-fluorescence bronchoscopy (AFB), and OCT were performed at the lesion location. The main characteristics of OCT imaging for the differentiation between benign and malignant lesions and the prediction of the pathological classification of lung cancer in bronchial lesions were identified, and their clinical value was evaluated.

**Results:**

A total of 135 patients were included in this study. The accuracy of OCT imaging for differentiating between benign and malignant bronchial lesions was 94.1%, which was significantly higher than that of AFB (67.4%). For the OCT imaging of SCC, adenocarcinoma, and small-cell lung cancer, the accuracies were 95.6, 94.3, and 92%, respectively. The accuracy, sensitivity, and specificity of OCT were higher than those of WLB. In addition, these main OCT image characteristics are independent influencing factors for predicting the corresponding diseases through logistic regression analysis between the main OCT image characteristics in the study and the general clinical features of patients (*p*<0.05).

**Conclusion:**

As a non-biopsy technique, OCT can be used to improve the diagnosis rate of lung cancer and promote the development of non-invasive histological biopsy.

## Background

Lung cancer is the leading cause of cancer deaths in the world (1–3), which is a serious threat to human health. At present, the “gold standard” for the diagnosis of lung cancer is histopathological results, which could be obtained by bronchoscopic biopsy, CT-guided lung biopsy, and surgical operation. These biopsy methods have high diagnostic accuracy; however, patients might suffer from complications such as bleeding, pneumothorax, and infection. Moreover, some of the biopsy methods are high risk, traumatic, and expensive (4, 5). In recent years, the “non-invasive histology” biopsy technology, which can effectively avoid the possible complications of tissue biopsy, has been developing by leaps and bounds. Recent studies (6–10) have also reported that non-invasive histological biopsy has high accuracy, sensitivity, and specificity for the diagnosis of lung cancer, in which optical coherence tomography (OCT) examination can achieve similar histopathological diagnosis without biopsy.

OCT is a high-resolution optical imaging technology, which has the characteristics of non-invasiveness, non-radiation, simple operation, and high repeatability. OCT integrates new technologies such as optics, supersensitive detection, and computer image processing. It utilizes low-energy near-infrared harmless light as the light source, and it detects the microstructure of biological tissue using optical interference principles. The resolution of the OCT image is 30 μm, and the depth of tissue that it detects is 3 mm. Mucous layer, submucosa, alveoli, glands, cartilage, and other structures of the bronchial wall are clearly presented in OC images, which are highly matched with histopathological images. OCT imaging could be applied to distinguish between benign and malignant bronchial lesions, predict the histological classification of lung cancer, and precisely detect small precancerous lesions that are unable to be seen with naked eyes.

In 1998, Pitris (11, 12) has confirmed the feasibility of OCT in human airway *in vitro*. Since then, studies (13–15) have proposed the OCT imaging features for different types of lung cancer with an accuracy of more than 80%. However, there are limitations with the present studies. First of all, the OCT images of adenocarcinoma, squamous cell carcinoma, and poorly differentiated cancer were only completed *in vitro* in these studies. It is known that the tissue degeneration and change of blood flow could largely affect the OCT images, leading to the inaccuracy of characteristics that were summarized. Second, the sample size of these studies was also small, so that the results need to be further verified. Third, the main OCT image features of small-cell lung cancer were only reported in case reports, which still need further verification (13). In addition, the OCT image features summarized in these studies were few and lacked unified image feature evaluation standards. Therefore, we aimed to explore the main OCT image features for the differentiation between benign and malignant lesions and for the prediction of pathological classification of lung cancer including central lung cancer and peripheral lung cancer and to evaluate their clinical value *in vivo*. The results of this study might be useful for the future application of OCT in the diagnosis, evaluation, and prognosis of lung cancer.

## Materials and methods

### Research population

This prospective study collected and analyzed the data of patients who underwent bronchoscopy biopsy and OCT examination at the Interventional Diagnosis and Treatment Center for Lung Cancer and Respiratory Diseases of the Chinese PLA General Hospital from February 2019 to December 2019. Prior to the study, all subjects had met the examination indications in the guidelines for the application of diagnostic flexible bronchoscopy in adults. This study was reviewed and approved by the Ethics Committee of CPLAGH (Ethics No. 2018-232-01). Patients were fully informed of the possible risks of the study and signed the informed consent form before the study began.

The admission criteria of this study were as followed: (1) voluntary participation and written informed consent signed by patients, (2) age ≥ 18 years old, (3) patients with routine diagnostic bronchoscopy through clinical evaluation, (4) normal ECG, and (5) adequate hematopoietic function of bone marrow and organs confirmed by blood examination. The exclusion criteria of this study were as follows: (1) patients with contraindication of bronchoscopy (such as respiratory failure and acute cardio-cerebrovascular events), (2) patients who refuse bronchoscopy because of physical reasons or personal wishes, (3) patients who are not suitable for bronchoscopy or OCT examination by researchers, (4) patients who cannot tolerate operation during bronchoscopy resulting in the uncompleted examination, (5) patients with unfound abnormal lesion during routine bronchoscopy or those who could not complete the biopsy, (6) patients who are participating in other clinical studies, (7) patients with poor compliance who are believed by the researchers to be unable to cooperate for the completion of OCT examination and follow-up, and (8) women who were pregnant.

### OCT

OCT system: The OCT system (Yongshida Medical Technology, Guangdong, China) has been approved by the FDA (K102599) for medical research and application. OCT system consists of image analysis system mainframe and aseptic removable probe. The probe of OCT is a cylindrical catheter with 1.7 mm in diameter and 150 cm in length, which is sealed with transparent sheath of 1 mm in length near its head and used for image scanning and acquisition. There is a flexible optical fiber axis in the sheath rotating at the speed of 600–1200 rpm; the working wavelength of the optical fiber passing the light source is 1300 nm and the frequency is 50kHz, the image acquisition speed is 10 frames per second, the axial and longitudinal resolution is 15 and 25 μm, respectively, and the detection depth of gray scale and color mode is 3 mm.

### Procedure

In this study, the relevant bronchoscopic procedures were performed by a respiratory physician with 7 years of experience in respiratory endoscopic diagnosis and treatment, including preoperative evaluation, preparation, and anesthesia. The respiratory endoscopic doctors completed the capability training on OCT examination following the learning curve prior to the formal inception of this study.

The respiratory endoscopic doctors had examined bronchial lesions with white-light bronchoscopy (WLB), auto-fluorescence bronchoscopy (AFB), and OCT successively after the completion of preoperative anesthesia. Routine tissue biopsy was performed on the same location after OCT examination, and the biopsy samples were fixed in 10% formalin for subsequent histopathological examination. If the lesion failed to be found or biopsied during the bronchoscopy, the OCT examination will not be performed, and the subsequent operation such as EBUS-TBNA and BAL will be carried out routinely by the doctor. The patients were followed up by telephone or outpatient service on the 3rd and 7th day after the examination, and adverse events and auxiliary examination results were recorded. The data of all patients were recorded in detail as shown in the table of case report.

### Histopathology

After the OCT examination, the tissue specimens were immediately fixed in 10% formalin, processed and sliced according to the standard histological procedure, and stained with HE. Two independent pathologists analyzed histological sections of each subject according to the lung tumor classification strategy proposed by WHO, IASLC, ATS, and ERS (16, 17). Adenocarcinoma, SCC and small-cell lung cancer were diagnosed by micro-endoscopy (magnification: 10–20).

### Analysis on the images of WLB, AFB, and OCT

The images of WLB and AFB were analyzed by respiratory endoscope operators without knowing the histopathological results, whereas OCT images were analyzed by the professional analysts and three clinicians with professional training experiences. The clinical values of WLB, AFB, and OCT in differentiating benign bronchial lesions from malignant ones and predicting different types of lung cancer were further evaluated based on the histopathological results.

### Statistics

SPSS23.0 statistical software was used for statistical analysis. The measurement data that accord with the normal distribution was expressed by X ± *SD* and the abnormal distribution by the median value. The counting data were expressed by the percentage. Paired *t*-test was used to compare the intra-group data in accordance with normal distribution. Independent sample *t*-test was used to compare inter-group data. Paired rank sum test was used to compare intra-group data in accordance with abnormal distribution, and rank-sum test for independent sample was used to compare inter-group data.

## Results

### Clinical information

A total of 135 patients were enrolled in this study. One hundred five (77.78%) were men and 30 (22.22%) were women; the mean age of the patients was 59.25 ± 10.35 (27–78) years. Ninety-eight (72.59%) patients had a history of smoking. All patients were examined by bronchoscopy biopsy and OCT successfully under local or basic anesthesia, including 126 (93.33%) cases with local anesthesia and 9 (6.67%) cases with basic anesthesia. There were 66 cases with left bronchial lesions and 69 cases with right bronchial lesions. Histopathological results of bronchial lesions included 30 (22.22%) cases with benign lesions and 105 (77.78%) cases with malignant lesions. There were 52 (49.52%) cases of lung squamous cell carcinoma including 19 (18.10%) cases with lung adenocarcinoma and 34 (32.38%) cases with small-cell lung cancer. The average time of OCT examination was 2.8 ± 1.6 min (**Table 1**). There were no adverse events directly related to OCT examination in this study.

**Table 1:** The clinical data of patients.

Characteristic	Values
Gender——n (%)	
Male	105 (77.78)
Female	30 (22.22)
Age——Years	59.25±10.35
Smoking——n (%)	
Yes	98 (72.59)
No	37 (27.41)
Anesthetic method——n (%)	
Local anesthetic	126 (93.33)
Basic anesthetic	9 (6.67)
Location of lesion——n (%)	
RMB	4 (2.96)
RUB	20 (14.81)
RISB	11 (8.15)
RMIB	12 (8.89)
RLB	19 (14.07)
LMB	10 (7.41)
LUB	10 (7.41)
LSLB	9 (6.67)
LLB	5 (3.70)
LLLB	35 (25.93)
Histopathology——n (%)	
Benign	30 (22.22)
Inflammation of mucosa	23 (76.67)
Granulomatous inflammation	3 (10)
Necrotizing inflammation	4 (13.33)
Malignant	105 (77.78)
SCC	52 (49.52)
adenocarcinoma	19 (18.10)
SCLC	34 (32.38)
Time (min)	
Total	18.6±6.5
WLB	8.7±1.2
AFB	7.1±1.4
OCT	2.8±1.6

### The image features and clinical values of OCT, WLB, and AFB

**Figure 1:**
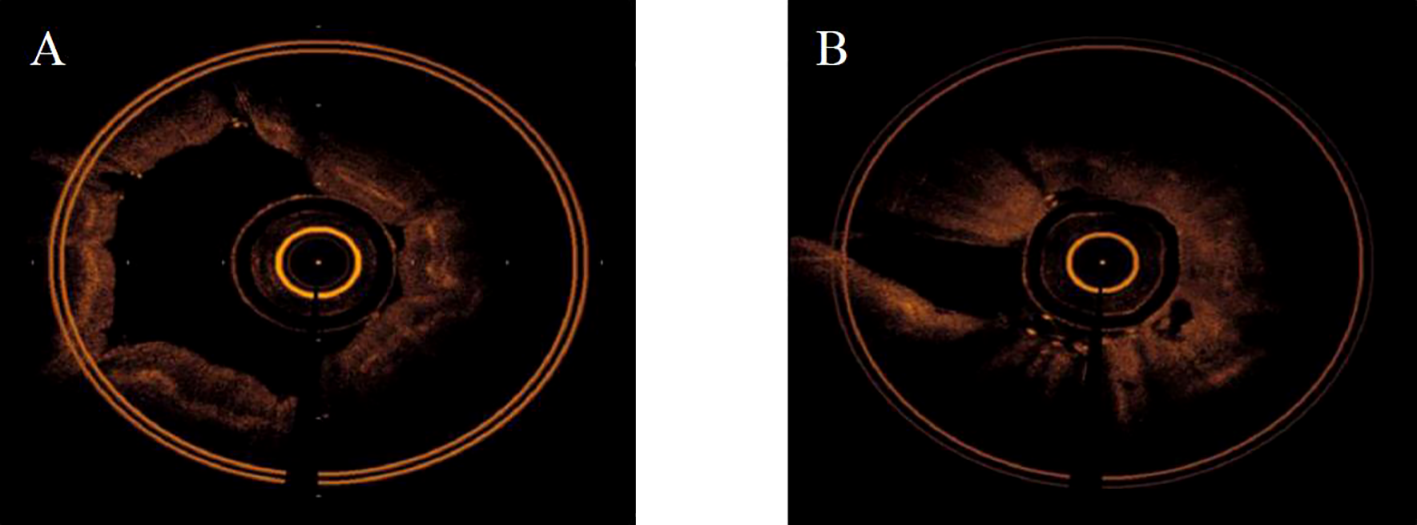
The main OCT feature of benign and malignant bronchial lesions. **(A)**: benign bronchial lesions. **(B)**: malignant bronchial lesions.

**Figure 2:**
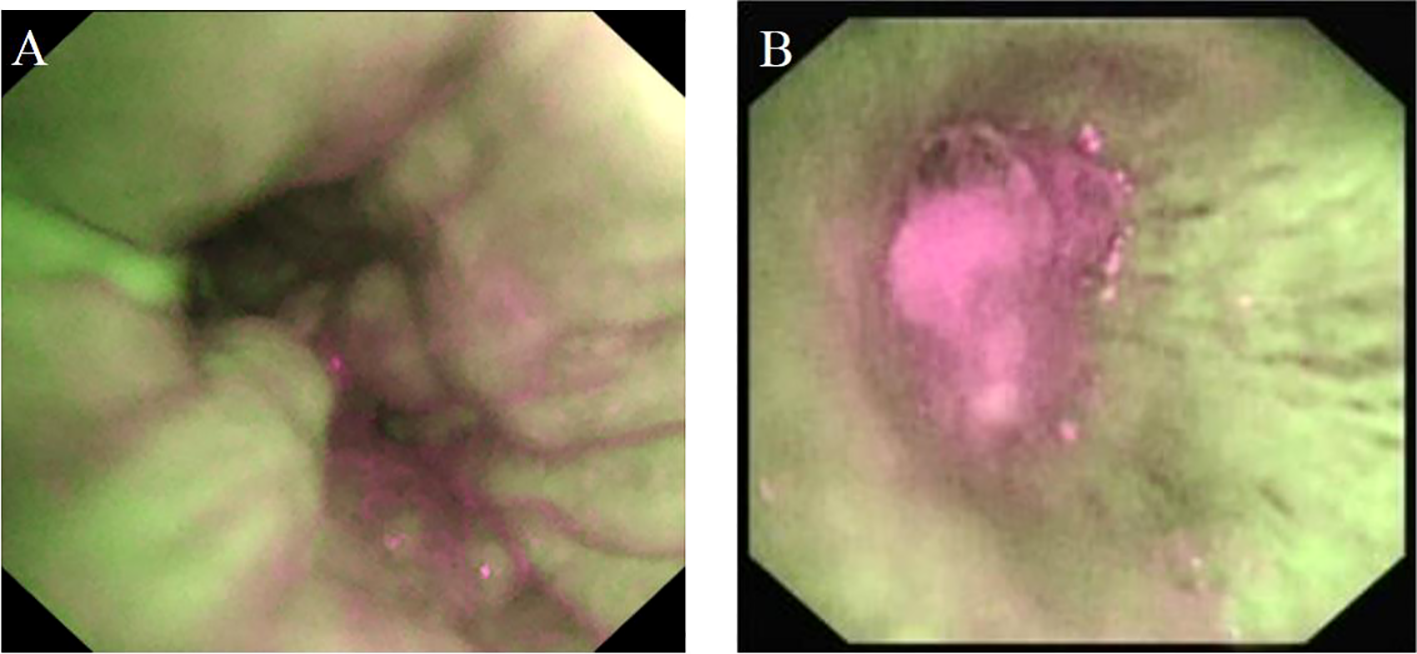
The AFB image feature of benign and malignant bronchial lesions. **(A)**: benign bronchial lesions. **(B)**: malignant bronchial lesions.

The main OCT image features of benign bronchial lesions was the integrity of normal structure with or without mucosal edema, whereas the image of malignant bronchial lesions was the destruction of normal structural layers including mucous layer, submucosa, and adventitia (**Figure 1**). The accuracy, sensitivity, and specificity of distinguishing benign from malignant bronchial lesions based on the main OCT image features were 94.1%, 97.1% and 83.3%, respectively. Receiver operator characteristic curve (AUC) = 0.902 ± 0.041 (95% Cl = [0.821, 0.983], *P* < 0.001). The AFB images of benign bronchial lesions are green, whereas those of malignant bronchial lesions are pink (**Figure 2**). The accuracy, sensitivity, and specificity of distinguishing benign from malignant bronchial lesions through AFB images were 67.4, 78.1, and 30%, respectively. AUC = 0.540 ± 0.061 (95% Cl = [0.421, 0.660], *P* < 0.001). It can be seen that the accuracy, sensitivity, and specificity of OCT in differential diagnosis of benign and malignant bronchial lesions are higher than those of AFB.

**Figure 3:**
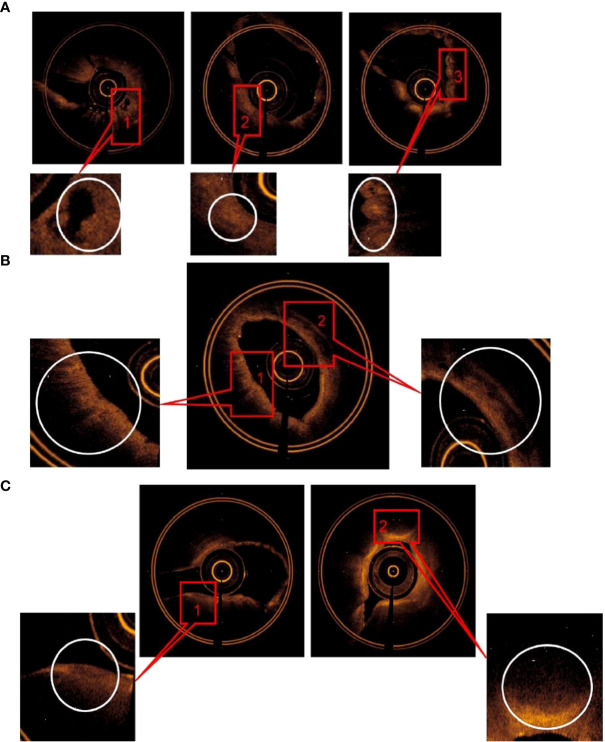
**(A)** The main OCT image features for predicting SCC: (1) “Cyst-like” structure, (2) “round” or irregularly shaped” high signal nests, with or without low-density shadow, (3) Scaly protuberance of the epithelial layer, and a darkened gray level of the submucosa layer was found. **(B)** The main OCT image features for predicting adenocarcinoma: (1) “Round” or “angulated” poor signal structures was found in mucous layer, (2) continuous low density shadow was found in submucous layer. **(C)** The main OCT image features for predicting SCLC: (1)Low signal lines, which resembled “fracture line”, (2) a significantly thickened lamina propria with a darkened gray level of the submucosa layer w2as foundcontinuous low density shadow was found.

The main OCT image features of squamous cell carcinoma include (1) “cyst-like” structure; (2) “round” or “irregularly shaped” high-signal nests, with or without low-density shadow; and (3) scaly protuberance of the epithelial layer and a darkened gray color of the submucosa layer. The accuracy of image features (2) for predicting squamous cell carcinoma was 95.6%. The sensitivity and specificity were 96.2 and 93.8%, respectively. AUC = 0.964 ± 0.025 (95% Cl = [0.916, 1.000], *P* < 0.001). The specificity was 100% by combining the image features (1) and (3), but the sensitivity was reduced to 75% (**Figure 3A**).

The main OCT image features of adenocarcinoma include (1) “round” or “angulated” poor signal structures in mucous layer and (2) continuous low-density shadow in submucous layer. The accuracy was 94.3% by combining image features (1) and (2) for predicting adenocarcinoma, the sensitivity and specificity was 89.5 and 100%, respectively, and AUC = 0.947 ± 0.042 (95% Cl = [0.865, 1.000], *P* < 0.001) (**Figure 3A**).

The main OCT image features of small-cell lung cancer include (1) low signal lines, which resembled “fracture line,” (2) a significantly thickened lamina propria with a darkened gray color of the submucosa layer. The accuracy for predicting small-cell lung cancer was 92% by combining OCT image features (1) and (2); the sensitivity and specificity was 91.2 and 93.8% respectively; AUC = 0.939 ± 0.034 (95% Cl = [0.872, 1.000], *P* < 0.001) (**Figure 3C**).

**Figure 4:**
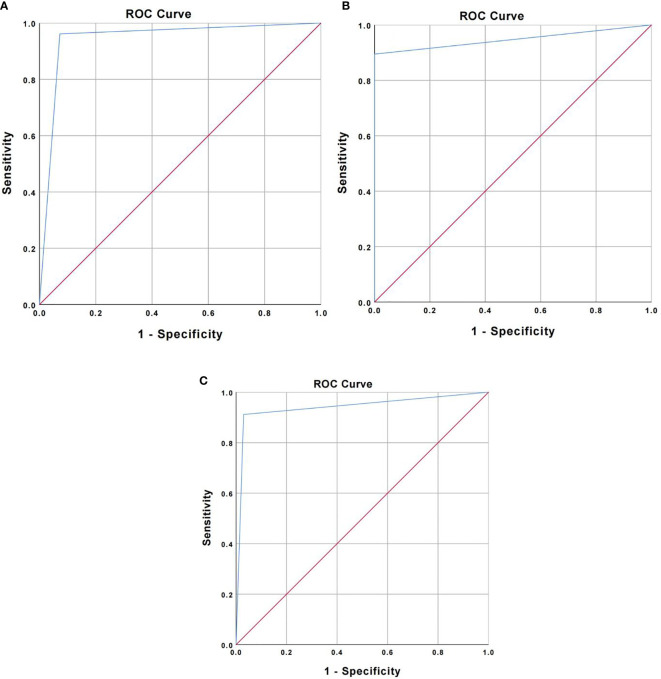
**(A)** ROC curve for verifying the accuracy of major OCT image features of squamous cell carcinoma. **(B)** ROC curve for verifying the accuracy of major OCT image features of adenocarcinoma. **(C)** ROC curve for verifying the accuracy of main OCT image features of small cell lung cancer.

The accuracy was verified by incorporating the main OCT image features for the three types of lung cancer into the total sample size and drawing ROC curves, respectively. The AUC was 0.945 ± 0.023 for squamous cell carcinoma (95% Cl= [0.900, 0.989], *P* < 0.001), 0.947 ± 0.041 for adenocarcinoma (95% Cl = [0.866, 0.989], *P* < 0.001), and 0.941 ± 0.030 for small-cell lung cancer (95% Cl = [0.882, 1.000], *P* < 0.001). Therefore, the main OCT image features for the three types of lung cancer in this study have been verified with high accuracy (**Figure 4**). In addition, the study showed that the accuracy of WLB in predicting squamous cell carcinoma, adenocarcinoma, and small-cell lung cancer was about 70%, which was significantly lower than that of OCT in predicting the pathological classification of lung cancer.

**Figure 5:**
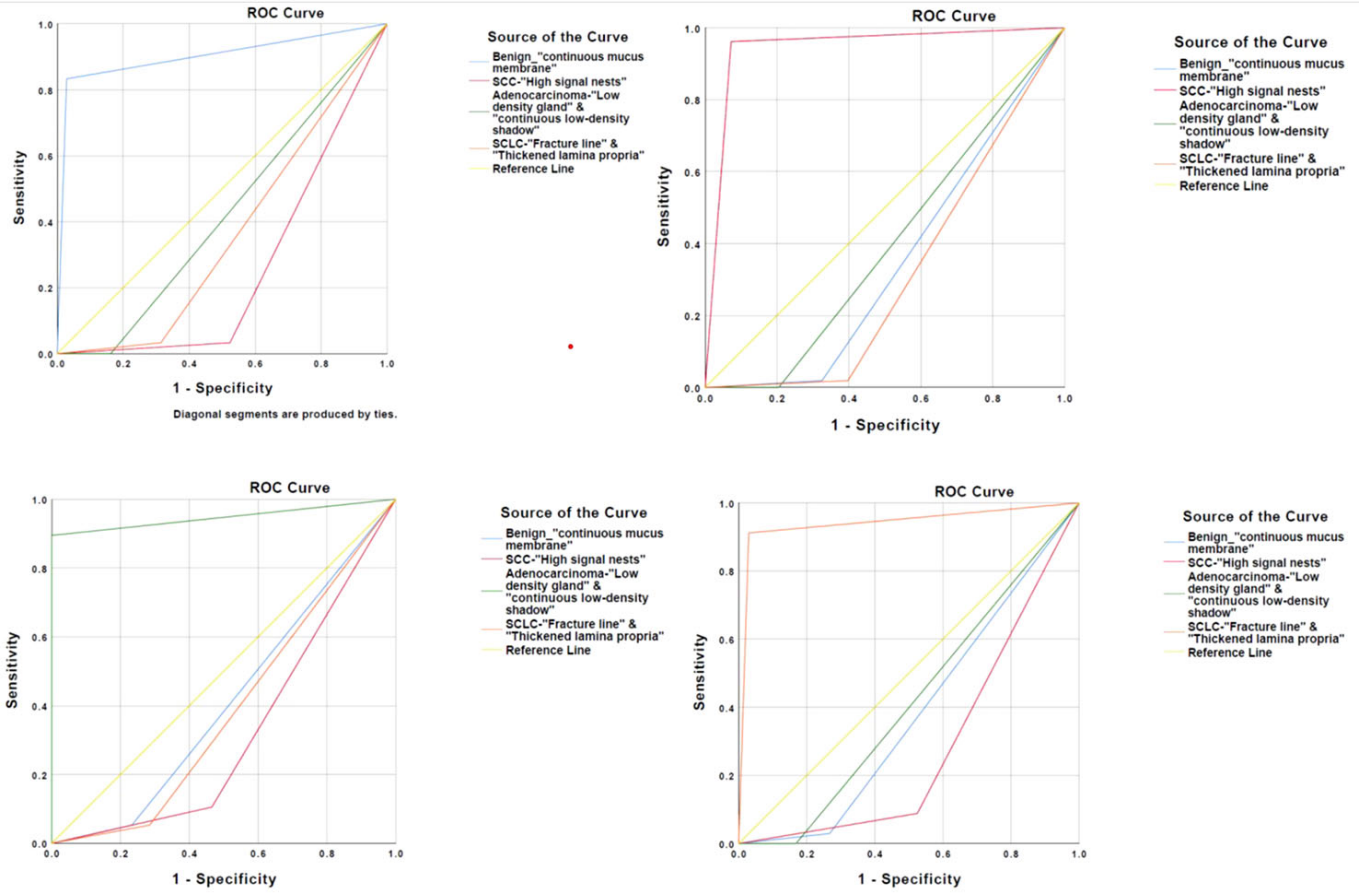
Four main OCT image characteristics have a high degree of differentiation to predict the corresponding disease.

We have conducted a further statistical analysis on the main OCT image features for benign and malignant lesions and for the three types of lung cancer by combining with the total sample size and drawing ROC curves to evaluate its accuracy for the diagnosis of bronchial lesions. The AUC was 0.902 ± 0.023 for benign bronchial lesions (95% Cl= [0.821, 0.983], *P* < 0.001), 0.945 ± 0.041 for squamous cell carcinoma (95% Cl= [0.900, 0.989], *P* < 0.001), 0.947 ± 0.041 for adenocarcinoma (95% Cl= [0.866, 1.000], *P* < 0.001), and 0.941 ± 0.030 for small-cell lung cancer (95% Cl= [0.882, 0.983], *P* < 0.001) (**Figure 5**). In addition, these main OCT image features are independent influencing factors for predicting the corresponding diseases through constructing Logistic regression analysis between the main OCT image characteristics in the study and the general clinical features of patients (**Table 2**). Therefore, the main OCT image that features each has shown a good differentiation from the other three features in predicting the corresponding diseases, that is, different OCT image features have specific corresponding diagnostic significance.

**Table 2:** The logistic regression analysis of the general clinical features and main OCT image characteristics in the differentiation of benign and malignant bronchial lesions and histopathological classification of lung cancer.

OCT	B	S.E.	Wald	p	OR
					(95% CI)
Distinguish between benign and malignant	5.136	0.764	45.232	<0.001	170
					(38.058, 759.374)
SCC-"High signal nests"	5.771	0.836	47.6	<0.001	320.833
					(62.272, 1652.987)
Adenocarcinoma-"Low density gland" & "continuous low-density shadow"	4.642	1.06	19.162	<0.001	103.765
					(12.983, 829.312)
SCLC-"Fracture line" & "Thickened lamina propria"	5.822	0.842	47.795	<0.001	337.556
					(64.796, 1758.491)

## Discussion

OCT is a newly developed technology with real-time imaging *in vivo*. Researches about OCT have been conducted in various fields of medicine (18), especially in ophthalmology, cardiovascular, and digestive tract diseases. OCT outperformed CT and ultrasound with its high resolution and non-radiation process, indicating its potential clinical application.

At present, OCT has been applied in respiratory diseases such as COPD, bronchial asthma, and lung cancer based on the principle that tissue images can be constructed by computer processing after infrared scattering, and the microstructure of bronchial wall can be recognized in real time. Unlike ultrasound, light waves of OCT do not need liquid medium for propagation, indicating that OCT is more suitable to be used in airway imaging. Moreover, OCT does not require a catheter to contact the tissue, which in turn minimizes adverse reactions that are commonly seen in invasive examination procedures. There is also no risk of exposing to infrared light in a short period of time. In addition, the OCT catheter probe reaches the end of the bronchoscope through the working channel of the bronchoscope and then is further sent to the lesion. During the operation, there might be adverse reactions caused by the catheter touching the bronchial wall or the lesions, so scholars have listed OCT examination as one of the “minimally invasive” examinations (19). The adverse events were consistent with the above study, and there were no adverse reactions directly related to OCT examination. Only several cases of mild bleeding or cough were noticed. The adverse reactions were improved and did not affect the continuation of OCT examination after short observation or local administration of a small amount of hemostatic drugs. In this study, the analysts for OCT image features consisted of a professional OCT image analyst and a trained respiratory physician, pathologist, and thoracic surgeon, which were of great significance for judging the histological features of lung cancer, evaluating OCT image features and imaging artifacts, and assessing whether OCT imaging can be evaluated in real time during the examination. In addition, although the real-time scanning location of OCT catheter was close to that of biopsy, it was not completely consistent *in vivo*, so the OCT images could not match the images of tissue pathological sections under microscope, and the one-by-one comparative analysis was not available; therefore, the clinical value of OCT images was evaluated based on the results of histopathology. The OCT examination takes only a few minutes, and the tissue microstructure of bronchus such as mucous membrane, submucosa, cartilage, and adventitia were imaged with high resolution without contact with the bronchus in our study; these data are consistent with the findings in other literatures (20, 21). It is worth noting that it is recommended to increase local topical anesthesia for the target lesions and its adjacent bronchi prior to OCT examination, which is useful to increase the tolerance of patients, and reduce the possibility of OCT catheter touching the bronchial wall and lesions, so as to prevent cough stimulation from affecting the imaging effect and destroying the lesions.

The traditional method of respiratory endoscopy for distinguishing benign bronchial lesions from malignant ones is to identify with the naked eyes through the endoscopic images formed in the process of WLB or AFB. WLB has significantly increased the image resolution and improved the ability to distinguish the nature of bronchial lesions by using electronic bronchoscope instead of fiberoptic bronchoscope. However, it is still difficult for experienced bronchoscopic operators to identify subtle mucosal lesions (22, 23). Fluorescence bronchoscopy has been proved to be highly sensitive in differentiating benign and malignant endobronchial lesions, but it has a high false positive rate due to bronchial mucosal inflammation and bleeding. Low specificity is another problem for fluorescence bronchoscopy (24, 25), which is further confirmed in our research results. Former studies (12, 13, 26) have confirmed that OCT can distinguish normal bronchial tissue from tumor lesions with the principle that the mucous layer and submucosa of bronchial wall thickened in different stages of cancer; however, no study had summarized and proposed the main OCT image features in distinguishing benign bronchial lesions from malignant ones. In our study, for the first time, we found that whether the mucosal layer is edematous and the normal structural layer is destroyed are the main OCT image features in distinguishing between benign and malignant bronchial lesions. The accuracy of the main OCT image features in distinguishing benign and malignant bronchial lesions was 94.1%, and the sensitivity and specificity were 97.1 and 83.3%, respectively, both of which were significantly higher than those of AFB. Therefore, comparing with AFB, OCT has more clinical value in distinguishing benign and malignant bronchial lesions. It is worth noting that the histopathological results of malignant lesions were invasive cancer in our study, whereas OCT images of precancerous lesions and carcinoma *in situ* could also show the integrity of normal tissue structure, which was reported in early studies. However, there was a significant difference between benign and malignant bronchial lesions in OCT images in our findings. Therefore, the differentiation between bronchial benign lesions and bronchial precancerous lesions or carcinoma *in situ* still needs more research and further exploration.

OCT has been used to assist the diagnosis and treatment of lung cancer with the development of interventional diagnosis and treatment of respiratory diseases. Many studies (14, 15, 27) have preliminarily reported that the accuracy of main OCT image features for distinguishing adenocarcinoma, squamous cell carcinoma, and poorly differentiated lung cancer *in vitro* was more than 82.6%. However, whether the OCT image features of tissue specimens *in vitro* can reflect the actual condition still needs to be further confirmed, because tissue degeneration and internal blood flow changes will occur in the excised specimens, and the effects of cough, spontaneous breathing, heartbeat, and secretions on the imaging results will not be truly reflected in the examination process. In our study, the clinical value of OCT in real-time diagnosis of lung cancer was evaluated for the first time, and the main OCT image features for predicting adenocarcinoma, squamous cell carcinoma, and small-cell lung cancer were proposed and verified. There are three main OCT image features with squamous cell carcinoma. The sensitivity of any image features in predicting squamous cell carcinoma was 100%, but the specificity was only about 31.2%. The specificity was 100% by combining the three image features to predict squamous cell carcinoma, but the sensitivity was very low. However, the main image features (2) showed high clinical value with the accuracy of 95.6%, and the sensitivity and specificity were 96.2 and 93.8%, respectively. The accuracy and specificity of the main OCT image features of squamous cell carcinoma, which was proposed by previous studies *in vitro*, were 82.6 and 87% (14), respectively. Therefore, the main OCT image features (1), (2), or (3) can be used as the preliminary screening image for the diagnosis of squamous cell carcinoma, and the final image features should be the main OCT image features (2) or the combination of features (1), (2), and (3). It is recommended to choose the combination of each (1) and (2) as the main OCT image features in predicting adenocarcinoma and small-cell lung cancer. The accuracy of the main OCT image features of adenocarcinoma was 94.3%; the sensitivity and specificity were 89.5 and 100%, respectively, which were significantly higher than those in the previous studies *in vitro* (80.3 and 88.6%). The main OCT image features of small-cell lung cancer were proposed by the study for the first time and initially showed high accuracy, sensitivity, and specificity. Therefore, compared with previous studies, the main OCT image features in the study can be used to predict the histological classification of lung cancer significantly. In addition, the sensitivity and specificity have been comparable with the results of studying esophageal and cardiovascular diseases for which the application of OCT is more mature (28–30) and the accuracy results by verification had significant statistical results (*P*<0.05). In addition, the study also found that the accuracy, sensitivity, and specificity of OCT in predicting the pathological classification of lung cancer were higher than those of WLB, so the clinical value should be affirmed. While the OCT images cannot be compared with the histopathological images one by one, therefore, the analysis of OCT images is affected by subjective factors to a certain extent, and the related research data need to be confirmed by more studies. The latest study (31) reports that the OCT catheter is integrated into the 19G puncture needle, which design is useful to highly match the location of OCT examination and aspiration tissue, and the OCT images are more matched with histopathological images, which is expected to obtain more OCT image features with reality and reliability. However, currently, the product is tested using animals; we are looking forward to the relevant findings for clinical research after the product is put on the market.

To sum up, OCT is very useful to clinicians in differentiating benign and malignant bronchial lesions and for the histological classification of lung cancer, especially for patients who are unable to perform tissue biopsy or cannot obtain accurate histological pathological results after biopsy. Meanwhile, it can also provide real-time imaging of the lesions during bronchoscopy and guide the location of bronchoscopy biopsy to improve the positive rate of biopsy and the diagnosis rate of lung cancer. We can even choose direct operation after evaluating the lesion by OCT examination for patients who was diagnosed as lung cancer clinically and have the chance for operation, and there is no need for bronchoscopic biopsy so that the lung cancer metastasis caused by biopsy can be avoided. In addition, studies (32, 33) have suggested that OCT is also helpful in the treatment of lung cancer including auxiliary airway stent implantation, APC, cryosurgery, and other interventional therapy and to explore the relationship between OCT image features and gene mutations in patients with lung cancer who cannot be biopsied because of high risk of secondary biopsy after targeted drug resistance. However, the findings in the studies were still preliminary. It is expected that more research findings will form a sufficient basis to support its clinical application. It is worth noting that artificial intelligence has been widely used in the diagnosis and treatment of lung cancer in recent years, and OCT will be combined with artificial intelligence in the diagnosis and treatment of lung cancer, but there are still few OCT image features that were found so far, which is not enough for mechanical learning and deep learning. However, the development of follow-up research and the establishment of the database will carry out mechanical learning with massive image features and establish a basic model according to the learning results, and deep learning combined with basic clinical features is conducive to the automation, standardization, and individualization of OCT in the diagnosis and treatment of lung cancer.

Although the findings of the study are quite encouraging, the accuracy, sensitivity, and specificity are not sufficient to support OCT as a complete substitute for tissue biopsy. A study (34) reported that the histopathological results of specimens obtained by FNA were consistent with the pathological results of resection specimens, including 96.2% for adenocarcinoma and 84.7% for squamous cell carcinoma. There are differences between the accuracy of the main OCT image features of lung adenocarcinoma in our study and the above findings, which might due to some important factors that could affect the OCT process. For example, we mainly analyzed the OCT image features of the most common primary lung cancer, but the histological classifications of lung cancer diverses and the OCT image features of some rare lung cancer and metastatic lung tumors still need to be further explored. It has been reported that only 32% of poorly differentiated cancers can be accurately classified by histopathological method (34–36). With the rapid development of targeted therapy in the field of lung cancer treatment in recent years, it may be necessary to perform multiple tissue biopsies for gene detection to evaluate the targeted drug resistance of lung cancer and guide the next step of treatment (37–39). This situation makes other non-invasive tissue biopsy techniques including OCT difficult to completely replace tissue biopsy in a short time. In addition, the resolution of OCT cannot fully reach the level of the microscope, and adjustable factors such as lens selection, focal length adjustment, and high power lens field of view of the microscope cannot be achieved by OCT temporarily. Some factors affecting OCT imaging include spontaneous respiration, heartbeat, blood, airway secretions, calcification, fibrosis, and so on; more advanced technologies need to be developed for data acquisition and automatic image processing to improve the authenticity of airway images and standardize the clinical application of OCT images (40, 41).

## Conclusions

In summary, the safety and effectiveness of OCT were confirmed by our study; however, the current findings are not sufficient to support OCT as a complete substitute for tissue biopsy. Nevertheless, optical signals have stronger ability to penetrate tissues with the further development of OCT technology, and the images will have higher resolution and better matching with histopathological images after reducing the influencing factors. It is expected to carry out larger sample size research in the future including early screening of lung cancer and diagnosis of peripheral lung cancer to find more OCT image features of clinical significance. Big data integrate the clinical data of patients with OCT image features and combine with the artificial intelligence, which will play an important role in the clinical application of OCT for the diagnosis and treatment of lung cancer.

## Data availability statement

The raw data supporting the conclusions of this article will be made available by the authors, without undue reservation.

## Ethics statement

The local institutional review board approved this study (Ethics No. 2018-232-01). This study is registered at Chinese Clinical Trial registry with identifier ChiCTR1900021466. The patients/participants provided their written informed consent to participate in this study. Written informed consent was obtained from the individual(s) for the publication of any potentially identifiable images or data included in this article.

## Author contributions

LC conceived this study and drafted the manuscript. QZ and ZY collected the clinical data, interpreted the results, and wrote the manuscript. ZL, WZ, HY, MZ, YX, YJ, MG, CZ participated in data collection and critical revision of the manuscript. All authors contributed to the article and approved the submitted version.

## Funding

This study was supported by Research on the artificial intelligence-aided diagnosis and clinical decision-making system for ground glass nodules of lung (2020-1-5011), and the Research on the construction of big data platform for diagnosis and treatment with respiratory endoscopy (2019MBD-052).

## Acknowledgments

We thank all our colleagues at the Interventional Diagnosis and Treatment Center for Lung Cancer and Respiratory Diseases of the Chinese PLA General Hospital for making this study possible.

## Conflict of interest

The authors declare that the research was conducted in the absence of any commercial or financial relationships that could be construed as a potential conflict of interest.

## Publisher’s note

All claims expressed in this article are solely those of the authors and do not necessarily represent those of their affiliated organizations, or those of the publisher, the editors and the reviewers. Any product that may be evaluated in this article, or claim that may be made by its manufacturer, is not guaranteed or endorsed by the publisher.

## Abbreviations

SCC, Squamous cell carcinoma; SCLC, Small-cell lung cancer; OCT, Optical coherence tomography; CT, Computed tomography; WLB, White-light bronchoscopy; AFB, Autofluorescence bronchoscopy; EBUS, Endobronchial ultrasound; BAL, Broncho alveolar lavage; HE, Hematoxylin–eosin; WHO, World Health Organization; IASLC, International Association for the Study of Lung Cancer; ATS, American Thoracic Society; ERS, European Respiratory Society; COPD, Chronic obstructive pulmonary disease; APC, Argon plasma coagulation; FNA, Fine-needle aspiration.
